# CMR scanning of MR-conditional pacemakers: a 5-year single centre experience

**DOI:** 10.1186/1532-429X-17-S1-P184

**Published:** 2015-02-03

**Authors:** Francesco Papalia, Saidi A Mohiddin, Ceri Davies, Mark Westwood, Roshan Weerackody, Sarah Whittaker-Axon, Neha Sekhri

**Affiliations:** Barts Heart Centre, Barts Health NHS Trust, London, UK

## Background

The first MRI conditional pacemaker was introduced in 2008^1^. Though, they represent the minority of all devices implanted, their use has increased.

Similarly, CMR imaging in the UK has increased from just over 20,000 studies in 2008 to nearly 40,000 scans in 2010^3^. However, there is limited data describing the frequency, safety and diagnostic quality of CMR scans in patients with MR conditional devices.

Our aim was to describe device scanning in our clinical practice and identify reasons why MR conditional pacemakers were selected.

## Methods

We interrogated our CMR registry to identify patients with MR conditional pacemakers (2010 to august 2014) who had undergone CMR. We also determined the indications for device implantation and cardiac imaging.

Our scanning protocol involves meticulous checking of the devices and pacing leads to ensure they are MR conditional, programming them to MR safe mode, scanning using a pre-specified protocol and checking the devices post scanning ( sensing, lead integrity and threshold) by a trained pacing physiologist. A 90 minute slot is reserved for each patient.

## Results

A total of 556 patients received MR conditional pacemaker devices (Figure). Of these, 22 patients underwent a CMR scan, a single individual in 2010 increasing to nine in 2014. 21 patients had a Medtronic device and one, a Biotronic device.

**Figure 1:**
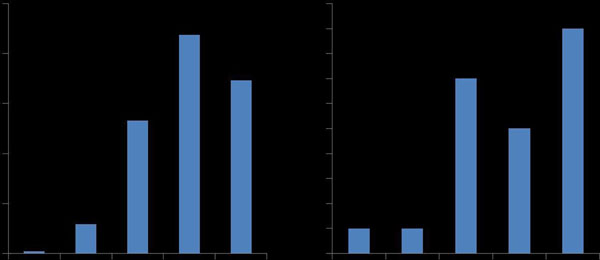
Figure 1

45% (n=10) of these 22 had more than one scan. The pre-implantation diagnosis included hypertrophic cardiomyopathy (9), sarcoidosis (3), myotonic dystrophy (1), an unspecified cardiomyopathy with conduction disease (3). The remainder had isolated conduction disease (6). 10 patients underwent an adenosine stress perfusion.

No patient or device complications were reported.

## Conclusions

In our experience, CMR scanning of patients with MR conditional pacemakers is safe under adequately controlled conditions following a prespecified safety protocol. We have experienced an increase in the implantation of MR conditional devices at our centre, which performs 3000-3500 CMR scans per year. Though, the number of scans in patients with an MR conditional device is very small relative to the total number of devices implanted, there is seen a steady increase over the past years. Further work is needed to understand the reasons behind decision made to implant MR conditional devices as it is likely more such patients will undergo MRI scanning in the future, which will impact on resources and service provision.

## Funding

N/a.

